# Transitioning from Cytology to HPV Test-Based Primary Cervical Screening in Canada: A Population-Based Survey of Women’s Screening and Information Preferences

**DOI:** 10.3390/curroncol33020095

**Published:** 2026-02-04

**Authors:** Ovidiu Tatar, Patricia Zhu, Shannon Salvador, Susie Lau, Jessica Ruel-Laliberté, Samara Perez, Emily McBride, Zeev Rosberger

**Affiliations:** 1 Lady Davis Institute for Medical Research, Jewish General Hospital, McGill University, Montreal, QC H3T 1E1, Canada; yue.y.zhu@mail.mcgill.ca; 2 Douglas Hospital Research Center, Montreal, QC H4H 1R3, Canada; 3 Department of Psychiatry, McGill University, Montreal, QC H3A 1A1, Canada; 4 Division of Gynecologic Oncology, Jewish General Hospital, McGill University, Montreal, QC H3T 1E2, Canada; shannon.salvador@mcgill.ca (S.S.); susie.lau@mcgill.ca (S.L.); 5 Department of Obstetrics & Gynecology, Division of Gynecologic Oncology, Université de Sherbrooke, Sherbrooke, QC J1H 5N4, Canada; jessica.ruel-laliberte@usherbrooke.ca; 6 Department of Oncology, McGill University, Montreal, QC H4A 3T2, Canada; samara.perez@mcgill.ca; 7 Research Institute of McGill University Health Centre, Montréal, QC H3H 2R9, Canada; 8 Psychosocial Oncology Program, Division of Supportive & Palliative Care, Cedars Cancer Centre, McGill University Health Centre, Montréal, QC H4A 3J1, Canada; 9 Institute of Psychology, Psychiatry and Neuroscience, King’s College London, London SE5 8AF, UK; emily.mcbride@kcl.ac.uk; 10 Department of Psychology, McGill University, Montreal, QC H3A 1G1, Canada

**Keywords:** cervical cancer, screening, self-sampling, human papillomavirus, preference, information, education, best–worst scaling, ranking, survey

## Abstract

**Simple Summary:**

In Canada, the Pap test (cytology), used for decades for cervical screening, is being replaced by human papillomavirus (HPV) testing, which can detect pre-cancer more sensitively and allows longer screening intervals. To support this transition, we conducted a national survey to understand women’s preferences for screening methods, starting age, screening intervals, and how screening information should be communicated. We found that women prefer using the HPV test in conjunction with the Pap test, starting screening at age 21, and maintaining screening intervals every three years. They also prefer receiving information by email rather than by postal mail. Women who do not participate regularly in screening showed high preferences for self-sampling and preferred receiving the HPV test from a gynecologist rather than a family physician. Successful implementation of the new screening test requires tailored information and empowering women to use self-sampling to support optimal uptake and progress toward cervical cancer elimination in Canada.

**Abstract:**

**Background**: Canada’s cervical cancer elimination plan is challenged by suboptimal screening participation and rising incidence of cervical cancer over the past decade. Cytology, the primary cervical screening method in Canada, is being replaced with HPV testing, which offers superior sensitivity for detecting pre-cancerous lesions and supports initiating screening at age 25 or older and extending screening intervals to five years. Research has shown that women’s insufficient knowledge and negative attitudes toward HPV screening represent a significant barrier to screening uptake. **Methods**: We conducted a web-based national survey using Best–Worst Scaling (trade off utilities) to quantify women’s preferences for screening test modality, age of initiation, and screening intervals. We also assessed preferences for information sources, provider type, and communication methods. Underscreened individuals were oversampled. **Results**: Among adequately screened (N = 1778) and underscreened (N = 1570) individuals, preferences favoured co-testing (cytology plus HPV testing), initiating screening at age 21, and three-year screening intervals. Underscreened participants showed relatively higher preference for HPV self-sampling, and as opposed to adequately screened participants, preferred screening by a gynecologist rather than a family physician. Across groups, participants preferred receiving screening-related information and communication by email over postal mail. **Conclusions**: The misalignment between women’s preferences and current HPV test-based screening implementation plans requires immediate education interventions and modernized, user-preferred communication channels for cervical screening-eligible individuals in Canada.

## 1. Introduction

Once envisioned as a cancer on the brink of elimination, cervical cancer is now projected to persist *well beyond* 2050, even in high-income countries, as prevention gains have stalled and screening transitions have lagged behind expectations [1]. The World Health Organization (WHO) launched a global strategy to eliminate cervical cancer, centered on achieving “90–70–90” targets by 2030, meaning that 90% of girls are fully vaccinated with the Human Papillomavirus (HPV) vaccine by age 15, 70% of women are screened using a high-performance test by age 35 and again by age 45, and 90% of women identified with cervical cancer or pre-cancer will receive appropriate treatment and care [2]. In line with this mandate, the Canadian Partnership Against Cancer (CPAC) released its own *Action Plan for the Elimination of Cervical Cancer in Canada, 2020–2030* [3], which aims to achieve elimination by 2040 through three key pillars: improve vaccination coverage, implement high-risk HPV test-based primary screening, (hereafter referred to as HPV testing), and ensure timely treatment of pre-cancerous lesions and invasive cervical cancer. Although the age-standardized rate of new cervical cancer cases in Canada declined significantly over several decades, it has reversed its course and showed a significant upward trend, increasing by about 3.7% per year between 2015 and 2019 [4]. This increase in cervical cancer incidence has led to calls for urgent, coordinated political action to ensure equitable, fully funded, and community-trusted HPV vaccination and cervical screening across Canada [5,6].

Over the last decade, cervical screening has increasingly shifted from cytology-based screening (i.e., Pap test) to HPV testing. HPV testing offers superior sensitivity (over 90%) for detecting pre-cancerous lesions compared to cytology (50–70%), with a negative HPV result indicating a very low risk of future cervical cancer, supporting the safe extension of screening intervals from three to five years [7,8,9]. A key innovation of HPV testing is the option for self-sampling, which allows an individual to collect their own vaginal sample with accuracy comparable to clinician-collected samples [10]. Another possible screening approach is co-testing, where both HPV and Pap tests are performed at the same time. Furthermore, given that HPV infection is prevalent in younger sexually active women [11], and that most HPV infections in younger individuals clear naturally, this has prompted recommendations to raise screening initiation age from 21 to 25 or 30 years [12,13,14], reducing potential harms from over-screening and unnecessary treatment.

As of January 2026, the global shift toward HPV-based primary cervical screening is well underway, though recommendations vary by region. European guidelines recommend HPV testing as the sole primary screening method, starting at age 30 for average-risk populations, and explicitly advising against cytology-based programs [15]. A conditional recommendation is made to start screening at age 25 if effective triage strategies are in place for HPV-positive individuals. The latest United States Preventive Services Task Force recommendations include cytology alone every three years for women aged 21–29, with more flexible options for ages 30–65, including primary HPV testing (with self-collection), co-testing every five years, or cytology alone every three [16]. Guidelines in China are similar, recommending primary HPV testing as the preferred testing method, starting at age 25 every five years [17]. In Canada, cervical screening recommendations vary widely by province [18]: most provinces still rely on Pap tests, starting at age 21 in Nova Scotia, Newfoundland and Labrador, Manitoba, and Nunavut [19,20,21,22]; and at age 25 in Alberta, Saskatchewan, New Brunswick, and Yukon, all at a frequency of every three years [23,24,25,26]. Meanwhile, Prince Edward Island (PEI), Ontario, British Columbia (BC), and Northwest Territories (NWT) have adopted HPV testing as the primary method, typically starting at age 25 every five years (except NWT, every three years) [27,28,29], with BC and NWT also offering self-sampling. In Quebec, although an organized cervical screening program has not yet been implemented, HPV testing is available in select regions at age 25 every five years, while Pap testing continues at age 21 every 2–3 years elsewhere [30,31].

In countries that have implemented school-based HPV vaccination programs between 2007 and 2010 and achieved high coverage, cervical cancer incidence among women under the age of 30 has declined substantially [32,33,34,35]. These trends suggest that early initiation of routine cervical screening may offer limited additional benefit, and initiation of screening at age 30 can be reasonable where vaccination and screening coverage is high.

Although the clinical rationale for HPV test-based screening is clear, *international implementation experience shows that evidence alone does not ensure public acceptance* [6]. The initial transitions in Australia and the UK were met with strong public resistance, including petitions against extended screening intervals that garnered tens-of-thousands to over a million signatures [36,37]. Years after implementation, challenges to primary HPV test-based cervical screening in these countries remain, with studies demonstrating persistent barriers including discomfort with testing for a sexually transmitted infection, anxiety about longer intervals and older screening age, and limited understanding and knowledge of HPV testing and the new guidelines [38,39,40]. Together, these cases demonstrate that public attitudes and preferences often lag behind evidence-based policy, emphasizing the need for country-specific data to anticipate and address implementation barriers.

Meanwhile, successful implementation of HPV test-based screening without sustained resistance has also been documented. In Norway, HPV primary screening was introduced through a randomized implementation pilot in 2015, in which women aged 34–69 were assigned to HPV testing every five years or cytology every three years, while women aged 25–33 continued cytology-based screening [41,42,43]. Following three years of evaluation, HPV testing every five years was adopted as routine practice for women aged 34–69, and later extended to women 25–69 in 2023, with extended HPV genotyping implemented in 2025. This phased, evidence-based implementation approach demonstrated that careful planning, age-specific strategies, and risk-based management can facilitate successful transition and sustained integration of HPV test-based screening into existing programs.

Underscreened women, defined as those who have not received a Pap test in more than three years or an HPV test in more than five years, is a key priority population group [5,44]. Approximately 30% of cervical cancers are diagnosed in this underscreened group [45]. CPAC has identified several population groups facing disproportionate risk, including recent immigrants and women with lower income or education, among whom only about 60% meet screening guidelines [45,46,47]. Underscreened women often report persistent structural and psychosocial screening barriers such as embarrassment, lack of autonomy, confidentiality concerns, limited provider access, and stigma associated with STI-related testing [48,49,50,51,52,53,54,55,56].

In a Canada-wide survey conducted by our research team, in both adequately screened and underscreened women, we found that lower knowledge about HPV testing and negative attitudes (e.g., fear, embarrassment, or perceived pain) were associated with lower intentions to participate in screening. The same study showed that higher perceived barriers and concerns about performing self-sampling were associated with lower intentions to use self-sampling [52].

Furthermore, successful implementation also depends on understanding preferences for how screening programs are delivered. Studies have shown that a health care provider’s recommendation can significantly improve cancer screening rates [57,58]. Effective communication modalities, from invitation to delivery, are also key. Interventions using text messages have been shown to increase cervical screening rates [59], and women who use email to communicate with providers are more likely to be up-to-date with screening [60]. While research shows that addressing patients’ informational needs reduces anxiety and improves adherence [61,62], comprehensive data on these preferences remain limited in the Canadian context.

In Canada, our team was the first to identify a preference–policy mismatch. In a pilot study that included 503 adequately screened and 524 underscreened women using Best–Worst Scaling, we found that women preferred shorter intervals (3 years) and earlier initiation age (21 years old). Adequately screened women favoured Pap with HPV testing (co-testing) and underscreened women preferring HPV self-sampling [63]. Although co-testing is generally not recommended as a primary screening strategy in organized screening programs, examining preferences related to co-testing remains important for understanding women’s perceptions of safety and reassurance during transitions to HPV-based screening, rather than as endorsements of co-testing as a policy option. These discordant findings between policy and preference findings supported the need for a larger, nationally representative study to confirm and expand on these results to help guide future educational needs for the public concerning best methods of cervical cancer screening.

The objectives of this study were to assess preferences of adequately screened and underscreened women for the following: (1) cervical screening tests, screening intervals and ages of initiation; and (2) information, screening participation, and communication methods. The study aimed to provide Canadian jurisdictions with essential national evidence on women’s screening and information preferences and guide the equitable and scalable planning and implementation of HPV-based primary cervical screening.

## 2. Materials and Methods

### 2.1. Study Design and Participants

This study is reported in accordance with the STROBE guidelines for cross-sectional studies (see Appendix A). We used a cross-sectional design and a web-based survey, available in English and French, to collect data between August and September 2022. Recruitment was facilitated by Dynata, an international survey research firm, through its panel of Canadian residents. Individuals aged 21 to 70 years who had a cervix and no history of cervical cancer were eligible to participate. Data were collected as part of a larger research project aimed at understanding psychosocial correlates of Canadian women’s intentions to participate in HPV test-based primary cervical screening. The detailed project methodology has been published elsewhere [64]. We oversampled underscreened individuals, defined as those who had not received a Pap test in the past three years or had never been screened in their lifetime. Adequately screened was defined as having received a Pap test within the last three years. This was in line with the 2–3-year screening interval for Pap testing (cytology) in most Canadian provinces at the time of data collection [65].

### 2.2. Measures

The survey was programmed by Dynata (Toronto, ON, Canada), an international survey research firm that maintains large survey panels. Our survey did not allow participants to skip questions, which ensured complete data for all respondents who completed the survey. The questionnaire was pre-tested and adapted to ensure adequate understanding of the content, using cognitive interviews with seven screening-eligible individuals. The questionnaire began by assessing eligibility criteria and cervical screening history. Three informative statements were provided: one on HPV testing, one on HPV testing using self-sampling, and one comparing HPV testing and Pap testing. Respondents then completed validated scales measuring: (1) cervical cancer, HPV, and HPV testing knowledge; (2) intentions to participate in HPV test-based screening and self-sampling; and, (3) attitudes and beliefs related to HPV testing and self-sampling. Next, measures of preferences for screening interval, age of initiation, information, and communication preferences. Finally, sociodemographic questions and two open-ended questions about HPV testing and self-sampling were presented. Results of multivariable associations between knowledge, attitudes, sociodemographics and intentions to participate in HPV test-based screening, along with item-level analyses of knowledge, attitudes and beliefs scales, have been published elsewhere [48,52].

We used the Best–Worst Scaling (BWS) methodology to measure preferences for screening intervals (Domain A) and age of initiation (Domain B) for HPV test-based primary cervical screening (See Table 1). BWS is grounded in the concept of conjoint analysis, which posits that the utility (preference) of items involves a trade-off between the utilities of all items under investigation [66]. This method allows for identifying the relative importance of items, as respondents are asked to indicate the best (most preferred) and worst (least preferred) items. We applied Case 2 BWS, which involves trade-offs between attributes (i.e., Pap test, HPV test, co-testing with Pap and HPV test, and self-sampling) and their levels: 3, 5, and 10 years within Domain A, and 21, 25 and 30 years within Domain B. To construct the choice sets, we used a simple orthogonal main effect design to create nine questions for each domain [67]. Within each question, the attributes remained the same (e.g., HPV test), while their levels varied. Participants were instructed to select the best and the worst combination of each attribute and its corresponding level (sample questions for each domain are provided in Table 1).

Preferences for information related to cervical cancer and screening, communication methods, and sample collection were measured using a ranking methodology which preserves the concept of utility trade-offs among a set of items. Within each question, participants ranked items from 1 (most preferred) to 4, 5, or 6 (least preferred), depending on the number of items included in that question.

### 2.3. Statistical Analyses

For the BWS questions, we applied both counting and modelling analysis approaches. In the counting approach, we subtracted the number of times each attribute-level (and its corresponding attribute) was selected as the worst from the number of times it was selected as the best. For each attribute and its corresponding attribute-levels, a best-minus-worst score (BWs) was calculated by summing these differences across all observations, with higher scores indicating stronger preference. The standardized BWs (stdBWs) were calculated by dividing the BWs by the product of the number of times each attribute (nine times) and each attribute-level (three times) was presented across the nine questions and the total number of participants. For the modelling approach, the BWS datasets were configured according to the marginal model, which assumes that participants evaluate all attribute-levels within a question (for example in Domain A: 3 years, 5 years and 10 years) to select the most preferred option (best) and all three attribute-levels to select the least preferred option (worst) [67]. We used conditional regression modelling to estimate the log-odds of preferences for attributes and attribute levels relative to the reference categories. Results are reported as odds ratios (OR) with 95% confidence intervals (CI).

For the ranking data, descriptive statistics included calculating the proportion of respondents who ranked each item first and the mean rank of each item, with lower mean ranks indicating higher preference. To evaluate whether rankings differed from what would be expected under random assignment, we tested whether mean item ranks were statistically different from 2.5, 3.0, or 3.5 for questions that included four, five, or six items, respectively [68]. We applied the Plackett–Luce model with maximum likelihood estimation to obtain log-worth parameters for each item, with worths scaled so that a value of 1 represents an item of average preference within the choice set [68]. For each estimated worth (w), we calculated 95% confidence intervals, and the probability that the item would be ranked first. Worth values greater than 1 indicate that an item is preferred more than the average item in that question, whereas worth values less than 1 indicate lower preference. Each of the five ranking questions was analyzed individually.

Consistent with the main study objectives, data from adequately screened and underscreened participants were analyzed separately. Additional subgroup analyses were conducted based on the primary language spoken at home, age, ethnicity, length of time living in Canada, gender identity, and income. Within each subgroup (e.g., participants whose primary language spoken at home was English), we reported odds ratios and item worth, consistent with the analytic approach described for BWS and ranking data. All analyses were conducted using R statistical software, version 4.3.3 [69]. For creating the BWS choice sets we used the R packages “DoE.base” [70] and “support.BWS2” [71]. For conditional regression we used the “survival” package [72] and for modelling of ranking data we used the PlacketLuce package [73].

## 3. Results

**Figure 1:**
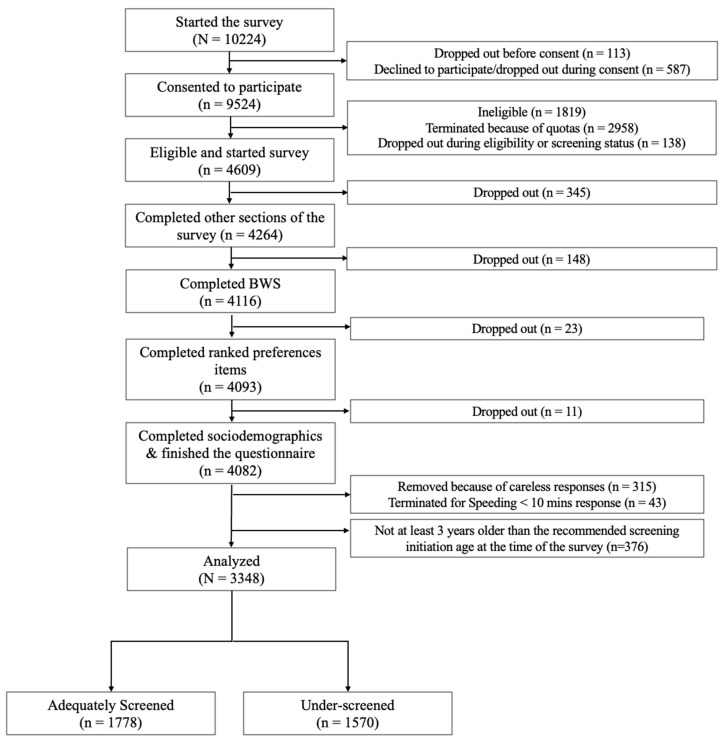
Participant Flow Diagram.

Of the 4609 participants who provided informed consent and met eligibility criteria, 4082 completed the questionnaire (88.6% retention rate). A total of 358 responses were removed following data cleaning that identified careless and inattentive responses. We used jurisdiction-specific age guidelines for screening initiation and included only women who were at least three years above the recommended starting age, i.e., older than 28 years in BC, Alberta, Nova Scotia, and PEI, and older than 24 years in all other jurisdictions. In total, we included 3348 women in the analyses, representing 1778 adequately screened and 1570 underscreened individuals, see Figure 1 Participant Flow Diagram. The sample characteristics are presented in Table 2.

### 3.1. Main Analyses

#### 3.1.1. Preferences for Cervical Screening Intervals

Descriptive analyses showed that among adequately screened participants, the most preferred attribute was receiving both the Pap and the HPV tests (co-testing, BWs = 3534, stdBWs = 0.22), whereas in underscreened participants, the most preferred attribute was self-sampling (BWs = 1990, stdBWs = 0.14). At the attribute level, adequately screened individuals most preferred receiving the co-test every three years (BWs = 3391, stdBWs = 0.64), while underscreened showed the highest preference for self-sampling every three years (BWs = 1468, stdBWs = 0.31) (Table 3).

Regression analyses revealed that in both groups, among attributes, preferences for the HPV test, co-test, and self-sampling were significantly higher than for the Pap test. Among the adequately screened, the largest effect size was observed for the co-test (OR = 2.29; CI = 2.21; 2.37), in contrast to the underscreened group, where the largest effect size was for self-sampling (OR = 1.97; CI = 1.90; 2.03). In both groups, attribute-level analyses (i.e., screening intervals) revealed significantly higher odds of preference for a three-year versus a five-year screening interval across all screening method options (adequately screened: 137% to 275% higher odds; underscreened 35% to 69% higher odds). Preferences for a 10-year screening interval versus a five-year interval were significantly lower in both groups (adequately screened: 63% to 78% lower odds; underscreened: 38% to 56% lower odds) (Table 3).

#### 3.1.2. Preferences for Age of Screening Initiation

Descriptive analyses of attributes revealed that, both in adequately screened and underscreened groups, the highest preference for receiving screening was with both the Pap and HPV tests (adequately screened: BWs = 4112, stdBWs = 0.26; underscreened: BWs = 1773, stdBWs = 0.13). Attribute-level analyses showed that in both groups the highest preference was for initiating cervical screening with the co-test at age 21 (adequately screened BWs = 1641, stdBWs = 0.31; underscreened: BWs = 880, stdBWs = 0.19) (Table 4).

Regression estimates showed that, in both groups, there was a significantly higher preference for initiating screening with the Pap test at 21 years versus 25 years (adequately screened: OR = 2.13, CI = 2.05; 2.22; underscreened: OR = 1.34, CI = 1.29; 1.39) and lower preferences for initiating screening with the Pap test at age 30 compared to 25 years (adequately screened: OR = 0.43, CI = 0.41; 0.44; underscreened: OR = 0.66, CI = 0.63; 0.68). Attribute-level estimates in both groups showed significantly higher preferences for initiating HPV test-based screening (i.e., HPV test alone, co-testing, or self-sampling) at age 21 or 25 versus 30 years. However, the effect size was higher for the comparison between 21 versus 30 years (adequately screened: 98% to 176% higher odds; underscreened 34% to 57% higher odds) than for the comparison between 25 versus 30 years (adequately screened: 9% to 28% higher odds; underscreened 9% to 20% higher odds) (Table 4).

#### 3.1.3. Information, Sample Collection, and Communication Preferences

As revealed by the Plackett—Luce models, participants in both groups showed the highest preference for receiving information about cervical cancer and screening from their provincial health agency (adequately screened: item worth (w) = 2.68, CI: 2.54; 2.83; underscreened: w = 2.42, CI = 2.29; 2.57), followed by receiving this information from a healthcare professional (HCP) (adequately screened: w = 2.24, CI: 2.12; 2.37; underscreened: w = 1.85, CI = 1.75; 1.96). In both groups, preference for receiving this information from social media was lowest (adequately screened: w = 0.21, CI: 0.20; 0.23; underscreened: w = 0.28, CI = 0.26; 0.30) (Question 1, Table 5 and Table 6).

In contrast to adequately screened, who most preferred receiving the HPV test as part of routine cervical screening from a family physician (w = 2.70, CI = 2.55; 2.87), underscreened had a higher preferences for receiving the HPV test from a gynecologist (w = 2.26, CI = 2.13; 2.40) than from a family physician (w = 1.73, CI = 1.63; 1.83) (Question 2, Table 5 and Table 6).

Regarding communication channels for receiving invitations and reminders to participate in routine cervical screening or to receive HPV test results, both adequately screened and underscreened showed the highest preference for email (e.g., for screening invitation among adequately screened: w = 2.68, CI: 2.54; 2.84; underscreened w = 2.78, CI: 2.62; 2.95), followed by postal mail (e.g., for screening invitation among adequately screened: w = 1.39, CI: 1.32; 1.47; underscreened w = 1.53, CI: 1.44; 1.62). Using an online portal to receive cervical screening invitations, reminders, or HPV test results was the least preferred option in both groups (Questions 3, 4, and 5, Table 5 and Table 6).

### 3.2. Additional Analyses

In all subgroups, preferences for receiving HPV test-based screening were higher than for the Pap test. In line with the results of the main analyses, the effect size for this comparison was highest for receiving both the HPV test and the Pap test (co-testing) (Appendix B and Appendix C). Across all subgroups and for all testing methods, preferences were significantly higher for being screened every three years compared to every five years, and lower for a ten-year interval compared to a five-year interval. In all subgroups, women preferred initiating screening with Pap at age 21 versus 25 and initiating screening with any HPV test-based screening method at age 21 or 25 versus 30 (Appendix B and Appendix C).

Participants whose primary language spoken at home was French most preferred receiving information related to cervical screening from the provincial public health agency (PHA) (w = 5.29) followed by international PHA (w = 1.99). In contrast, participants who primarily spoke English at home most preferred receiving this information from an HCP (w = 3.19), followed by a provincial PHA (w = 2.60) (Question 1, Appendix D). Those who primarily spoke at home languages other than English or French most preferred receiving information from the provincial PHA (w = 2.45) followed by an HCP (w = 1.96). Except for Indigenous people and individuals of European ethnicity, who most preferred receiving information from an HCP, preferences among other ethnic groups (e.g., Asian) followed the reverse pattern i.e., provincial PHA (Question 1, Appendix D).

In each subgroup, participants most preferred to receive the HPV test either from a family physician or from a gynecologist. Preference for receiving the HPV test from a family physician was highest among individuals who spoke English or French as their primary language at home, those aged 61 or older, North American Indigenous People, those of other North American ethnicity (e.g., Canadian, American), those of European ethnicity, participants who had lived in Canada for more than 10 years, participants who identified as women, and participants with a yearly household income over CAD 80,000 Canadian Dollars. Conversely, participants who did not primarily speak English or French at home, those younger than 60 years, individuals self-identifying as Asian, recent immigrants, gender diverse participants, and those with an annual household income below CAD 80,000 most preferred receiving the HPV test from a gynecologist (Question 2, Appendix D).

In all subgroups, email was the most preferred communication channel for receiving cervical screening invitations, reminders, or HPV test results. Preferences for postal mail as a second option were heterogenous, as participants who do not primarily speak English or French at home, those aged 30 years or younger, individuals of Asian ethnicity, and recent immigrants showed higher preferences for receiving text messages than postal mail. (Questions 3, 4, and 5, Appendix D).

## 4. Discussion

This national study used Best–Worst Scaling methodology to assess Canadian women’s preferences for both the clinical and operational components of cervical cancer screening. Our results offer key insights into the public acceptability of HPV-based primary screening and identify opportunities to align implementation strategies with women’s values and expectations. Clearly, we have demonstrated that important gaps exist between current national cervical screening guidelines and women’s preferences for testing that must be addressed through innovative and targeted interventions, including educational campaigns. Through carefully designed implementation strategies, Canada has the opportunity to overcome women’s lack of preparedness to transitioning from cytology to HPV testing.

### 4.1. Preferences for Screening Modality, Interval, and Initiation Age

The strong preference for co-testing (Pap and HPV) across screening groups demonstrates that many women value the reassurance of redundancy. Similar concerns were observed during Australia’s and the UK’s transitions to HPV testing, where public anxiety centered on the perceived loss of safety associated with the introduction of longer screening intervals and discontinuation of cytology [39,40,74]. These findings suggest that resistance to discontinuation of Pap testing is a response rooted in decades of messaging indicating that more frequent screening saves lives.

Our study confirms that women prefer initiating screening at 21 years and participating at three-year intervals regardless of the screening method. These preferences indicate a stable and widespread public sentiment that was in line with the findings of Rickford, Rogers, Halliday, Lamptey and Kola-Palmer [75], who found that U.K. women continued to prefer shorter intervals even after years of HPV-based screening. Therefore, it is important for new programs to be paired with transparent public education explaining the rationale and safety of extended intervals. In Canada, some provinces (e.g., Ontario), have even implemented Pap screening initiation at 25 years old. Evidence from communication studies suggests that reframing longer intervals as more “advanced” testing requires less frequent screening may help improve women’s attitudes and acceptance of these longer intervals [74,76]. The normalization and acceptance of HPV testing may take time to establish within the population.

Our study provides a detailed examination of underscreened women’s preferences, which is crucial for achieving health equity. In this group, aside from co-testing, HPV self-sampling was the most preferred method, reflecting the extant literature that self-sampling removes key structural (e.g., lack of time, accessibility to HCPs) and psychosocial barriers (e.g., embarrassment) [77,78,79], including underserved populations such as Indigenous communities [80]. Regarding implementation, a meta-analysis by Costa, Verberckmoes, Castle, and Arbyn [81] found that mail-in and face-to-face (e.g., community) approaches are the most effective interventions for increasing screening uptake amongst underscreened women. However, despite women’s willingness to self-sample, behavioural barriers remain. In prior work by our group, many women reported doubts about their ability to perform HPV self-sampling correctly [48], consistent with a systematic review identifying lack of confidence in self-sampling as a common reason for women’s preference for clinician-collected sampling [79]. To increase women’s confidence in collecting self-samples, behavioural interventions, such as brief demonstration videos, should be integrated with health service delivery to maximize screening participation.

### 4.2. Preferences for Information Sources, Providers, and Communication Methods

Women viewed provincial public health agencies (who in Canada are responsible for screening programs) as their most trusted information source, followed by HCPs. This finding reveals that as changes to screening programs are implemented, people rely on official health authorities to explain and justify new policies. Crucially, HCPs’ recommendation continues to be among the strongest predictors of screening adherence [82,83]. However, studies have found that HCPs’ practices do not always align with recommendations [84,85], reinforcing the importance of clinician education as part of policy rollout. Our findings further suggest that trust and preferred communication channels vary across population groups. Indigenous participants expressed the greatest preference for receiving information from HCPs and relationship-based communication, which aligns with calls for culturally safe, community-led screening, where local health-center staff and long-term engagement, instead of top-down public campaigns, are central [3,86]. Conversely, individuals who speak French at home showed lower preferences for HCP-based communication, likely reflecting Quebec’s systemic primary care access issues, where more than two million people lack a regular provider [87].

Preferences regarding provider type also reveal important system-level implications. Adequately screened women preferred to receive screening from their family physicians, whereas underscreened women, and populations typically underrepresented in screening (younger, lower income, recent immigrants, gender diverse), preferred gynecologists. This pattern likely reflects both a greater trust in consistent service and the structural reality that many Canadians lack a regular family doctor [87]. As such, expanding access points for HPV testing beyond primary care, including through sexual health clinics, colposcopy clinics, pharmacies, and community settings, may be key to reaching underscreened populations. For example, nurses in Quebec can perform cervical screening under the current provincial guidelines [88].

Regarding communication modalities, participants generally preferred email for invitations, reminders, and results, followed by postal mail. As most Canadian provinces still rely on mail-based invitations and reminders, with results typically delivered by phone, these findings point to a clear need to modernize communication practices within cervical screening programs. Meanwhile, exploratory analyses showed that younger, not speaking English or French, recent immigrants, ethnic, and gender diverse individuals, expressed stronger preferences for text messaging, aligning with evidence that text reminders can increase screening uptake [89]. Adopting a multimodal communication approach that allows users to choose their preferred communication channels may strengthen the reach, accessibility, and acceptability of cervical screening programs nationwide.

### 4.3. Strengths and Limitations

A key strength of this study is the use of Best–Worst Scaling, which provided deeper insight into the strength and trade-off of women’s screening preferences, extending beyond what conventional multiple-choice responses can capture. The large, pan-Canadian sample and purposeful oversampling of underscreened women strengthen external validity for screening-eligible populations, and addresses an important evidence gap for implementation planning.

Several limitations should be considered. First, as the survey was online, this may under-represent individuals without internet access or lower digital literacy. Given that 95% of Canadian adults had internet access and over 70% owned smartphones by 2022, this bias is likely modest [90]. Second, despite a large overall sample, the study was underpowered to analyze preferences within smaller subgroups such as Indigenous and gender-diverse individuals, highlighting the need for future participatory research with these communities. Third, preferences were elicited without providing detailed information on current guidelines or the evidence base (e.g., rationale for later initiation age and longer screening intervals with HPV testing). While this approach captures “unvarnished” preferences, it also indicates that responses may partly reflect familiarity with prior cytology-based messaging, rather than informed preferences under an HPV test-based program. Finally, stated preferences in a survey context may not fully predict real-world screening behaviour once HPV-based screening and communication strategies are implemented, and public and clinician education has occurred.

## 5. Conclusions

This national survey shows that Canadian women’s preferences for cervical screening currently diverge from key elements of HPV-based screening implementation, particularly regarding later initiation age and longer screening intervals. Preferences were strongest for more frequent screening, earlier initiation, and among many respondents, co-testing. Underscreened women expressed comparatively greater acceptability of self-sampling and a preference for specialist-based sampling.

Collectively, these findings highlight that in Canada, public acceptance will very likely lag behind policy change without proper education, messaging, and interventions. A dual-pronged educational approach is warranted: one targeting the public to build understanding and trust in HPV testing and self-sampling, and another targeting HCPs to ensure consistent, evidence-based recommendations. This effort also needs to be supported by provincial and federal public health initiatives working alongside clinicians as well as behavioural and implementation scientists. Tailored, culturally informed, and digitally adaptive communication strategies can support equitable participation. As Canada advances toward HPV-based primary screening, aligning implementation with women’s preferences will be central to ensuring programs that are not only scientifically sound but also trusted by women, patient-centered, and sustainable.

## Figures and Tables

**Table 1:** Best–Worst Scaling sample questions.

Question Two (Out of Nine) for Domain A (Screening Intervals)		
LEAST preferred		MOST preferred
	Cervical cancer screening with the **Pap test** every *3 years*	
X	Cervical cancer screening with the **HPV test** every *5 years*	
	Cervical cancer screening with **both the Pap test and the HPV test** every *10 years*	
	Cervical cancer screening with the **HPV test using HPV self-sampling** every *5 years*	X


**Question three (out of nine) for Domain B (screening initiation)**		
LEAST preferred		MOST preferred
X	Cervical cancer screening with the **Pap test** starting at age *21 years* old	
	Cervical cancer screening with **both the Pap test and the HPV test** starting at age *25 years* old	
	Cervical cancer screening with the **HPV test** starting at age *30 years* old	X
	Cervical cancer screening with the **HPV test using HPV self-sampling** starting at age *30 years* old	

For each question, participants selected one **least** preferred and one **most** preferred option (Examples marked with an “X”). The attributes are provided in bold, and the attribute levels are provided in *italics*.

**Table 2:** Sample Characteristics.

	Full Sample (N = 3348)
**Screening status**	
Adequately screened	1778 (53.1)
Underscreened	1570 (46.9)
**Primary Language, n (%)**	
English	2570 (76.8)
French	631 (18.8)
Other	147 (4.4)
**Age (years)**	
≤30	408 (12.2)
31–60	2161 (64.5)
≥61	779 (23.3)
**Ethnicity, n (%)**	
North American Indigenous ^1^	96 (2.9)
North American–Other ^2^	1526 (45.6)
European ^3^	1029 (30.7)
Asian ^4^	446 (13.3)
Other ^5^	251 (7.5)
**Living in Canada >10 years, n (%)**	
Yes	3135 (93.6)
No	213 (6.4)
**Gender identity, n (%)**	
Female/woman	3315 (99.0)
Gender diverse	33 (1.0)
**Household income, n (%)**	
CAD ≤ 39,999 ^6^	836 (25.0)
CAD 40,000–79,999	1119 (33.4)
CAD ≥ 80,000	1393 (41.6)

Note. ^1^ i.e., First Nations, Inuit, Metis; ^2^ e.g., Canadian, American, Ontarian, Quebecois, Acadian; ^3^ e.g., British, French, Western European, Eastern European; ^4^ e.g., West Central Asian, South Asian, East and Southeast Asian; ^5^ i.e., Caribbean (e.g., Cuban, Haitian, Jamaican), Latin, Central and South American (e.g., Mexican, Argentinian, Brazilian, Chilean), African (e.g., Central and West African, North African, Southern African), Oceania (e.g., Australian, New Zealander, Pacific Islander), and Other; ^6^ CAD denotes Canadian Dollar.

**Table 3:** Best–Worst Scaling results for screening interval.

	Adequately Screened (N = 1778)			Underscreened (N = 1570)		
	BWs	Std BWs	OR (95%CI)	BWs	Std BWs	OR (95%CI)
**Attributes**						
Pap test	−1908	−0.12	Ref	−2553	−0.18	Ref
HPV test	−431	−0.03	1.25 (1.21; 1.30)	−551	−0.04	1.35 (1.30; 1.40)
Pap and HPV test	3534	0.22	2.29 (2.21; 2.37)	1114	0.08	1.73 (1.67; 1.79)
Self-sampling	−1195	−0.07	1.06 ^†^ (1.02; 1.09)	1990	0.14	1.97 (1.90; 2.03)
**Attribute levels for *Pap test***						
Every 3 years	988	0.19	2.67 (2.57; 2.78)	−367	−0.08	1.35 (1.29; 1.40)
Every 5 years	−481	−0.09	Ref	−501	−0.11	Ref
Every 10 years	−2415	−0.45	0.33 (0.31; 0.34)	−1685	−0.36	0.60 (0.58; 0.63)
**Attribute levels for *HPV test***						
Every 3 years	1355	0.25	2.37 (2.28; 2.46)	388	0.08	1.39 (1.33; 1.44)
Every 5 years	−72	−0.01	Ref	44	0.01	Ref
Every 10 years	−1714	−0.32	0.37 (0.36; 0.39)	−983	−0.21	0.62 (0.60; 0.65)
**Attribute levels for *Pap and HPV test (co-test)***						
Every 3 years	3391	0.64	3.75 (3.59; 3.91)	1251	0.27	1.69 (1.63; 1.76)
Every 5 years	1569	0.29	Ref	877	0.19	Ref
Every 10 years	−1426	−0.27	0.22 (0.21; 0.23)	−1014	−0.22	0.44 (0.42; 0.46)
**Attribute levels for *self-sampling***						
Every 3 years	1187	0.22	2.60 (2.50; 2.70)	1468	0.31	1.61 (1.55; 1.68)
Every 5 years	31	0.01	Ref	1060	0.23	Ref
Every 10 years	−2413	−0.45	0.29 (0.28; 0.30)	−538	−0.11	0.49 (0.47; 0.51)

Note. Ref denotes reference category; BWs denotes best minus worst score; std BWs denotes standardized BWs. For attributes, std BWs was calculated by dividing BWs by 9 × 1778 for adequately screened and by 9 × 1570 for underscreened. For attribute levels std BWs was calculated by dividing BWs by 3 × 1778 for adequately screened and 3 × 1570 for underscreened. † denotes *p* < 0.01. All other odds ratios (OR) are significant at *p* < 0.001.

**Table 4:** Best–Worst Scaling results for age of screening initiation.

	Adequately Screened (N = 1778)			Underscreened (N = 1570)		
	BWs	Std BWs	OR (95%CI)	BWs	Std BWs	OR (95%CI)
**Attributes**						
Pap test	−2179	−0.14	Ref	−2692	−0.19	Ref
HPV test	−441	−0.03	1.28 (1.24; 1.33)	−556	−0.04	1.37 (1.32; 1.41)
Pap and HPV test	4112	0.26	2.46 (2.38; 2.55)	1773	0.13	1.93 (1.87; 2.00)
Self-sampling	−1492	−0.09	1.07 (1.04; 1.11)	1475	0.10	1.85 (1.78; 1.91)
**Attribute levels for *Pap test***						
21 years	584	0.11	2.13 (2.05; 2.22)	−411	−0.09	1.34 (1.29; 1.39)
25 years	−585	−0.11	Ref	−673	−0.14	Ref
30 years	−2178	−0.41	0.43 (0.41; 0.44)	−1608	−0.34	0.66 (0.63; 0.68)
**Attribute levels for *HPV test***						
21 years	1180	0.22	2.07 (2.00; 2.16)	382	0.08	1.39 (1.33; 1.44)
25 years	−94	−0.02	1.09 (1.05; 1.14)	−48	−0.01	1.09 (1.05; 1.14)
30 years	−1527	−0.29	Ref	−890	−0.19	Ref
**Attribute levels for *Pap and HPV test (co-test)***						
21 years	3146	0.59	2.76 (2.65; 2.88)	1355	0.29	1.57 (1.51; 1.63)
25 years	1641	0.31	1.14 (1.09; 1.18)	880	0.19	1.18 (1.13; 1.22)
30 years	−675	−0.13	Ref	−462	−0.10	Ref
**Attribute levels for *self-sampling***						
21 years	694	0.13	1.98 (1.90; 2.05)	995	0.21	1.34 (1.29; 1.39)
25 years	−100	−0.02	1.28 (1.23; 1.33)	789	0.17	1.20 (1.15; 1.25)
30 years	−2086	−0.39	Ref	−309	−0.07	Ref

Note. Ref denotes reference category; BWs denotes best minus worst score; std BWs denotes standardized BWs. For attributes, std BWs was calculated by dividing BWs by 9 × 1778 for adequately screened and by 9 × 1570 for underscreened. For attribute levels std BWs was calculated by dividing BWs by 3 × 1778 for adequately screened and 3 × 1570 for underscreened. All odds ratios (OR) are significant at *p* < 0.001.

**Table 5:** Results of rank analyses adequately screened (N = 1778).

Item	Descriptive Statistics		Plackett–Luce Model Estimates	
	Item Ranked First (%)	Mean Rank	Item Worth Compared to the Average Worth (*w*) (95% CI)	Probability of Highest Rank (%)
(Q1) prefer to receive information about cervical cancer and screening from ^§^				
International public health agency	11.9	3.49	1.03 (0.97; 1.08)	12.8
National public health agency	12.9	3.28	**1.27** (1.20; 1.33)	15.8
Provincial public health agency	26.4	2.31	**2.68** (2.54; 2.83)	33.4
Charities and non-profit organizations	1.3	4.40	**0.60** (0.57; 0.63)	7.5
Social media	2.4	5.07	**0.21** (0.20; 0.23)	2.7
A healthcare professional	45.1	2.45	**2.24** (2.12; 2.37)	27.9
(Q2) prefer to get the HPV test for my routine screening to prevent cervical cancer from ^§^				
Family physician	47.6	1.72	**2.70** (2.55; 2.87)	46.6
Gynaecologist	41.5	1.89	**2.11** (1.99; 2.23)	36.3
Nurse or nurse practitioner	10.4	2.75	**0.75** (0.71; 0.80)	13.0
Physician’s assistant	0.5	3.64	**0.23** (0.22; 0.25)	4.0
(Q3) prefer to receive the **invitation** to my routine screening to prevent cervical cancer by ^§^				
Postal mail	34.9	2.53	**1.39** (1.32; 1.47)	22.6
E-mail	38.5	1.95	**2.68** (2.54; 2.84)	43.7
Text message	7.8	3.24	**0.86** (0.82; 0.90)	14.0
Phone call	13.3	3.22	**0.83** (0.78; 0.87)	13.5
Online portal	5.5	4.06	**0.38** (0.36; 0.40)	6.2
(Q4) prefer to receive **reminders** about my routine screening to prevent cervical cancer by ^§^				
Postal mail	30.0	2.71	**1.19** (1.13; 1.26)	19.4
E-mail	39.1	1.94	**2.74** (2.59; 2.90)	44.6
Text message	13.8	3.01	1.01 (0.96; 1.07)	16.5
Phone call	12.1	3.22	**0.84** (0.80; 0.89)	13.7
Online portal	4.9	4.13	**0.36** (0.34; 0.38)	5.8
(Q5) prefer to receive the **results** of the HPV test by ^§^				
Postal mail	26.3	2.73	**1.22** (1.16; 1.29)	21.2
E-mail	31.6	2.10	**2.30** (2.19; 2.43)	40.0
Text message	6.2	3.48	**0.74** (0.70; 0.78)	12.8
Phone call	24.8	2.90	1.03 (0.98; 1.09)	17.9
Online portal	11.2	3.79	**0.47** (0.44; 0.49)	8.1

Note: ^§^ denotes significant Chi-square test (*p* < 0.001) showing non-random rankings. All estimates shown in bold are significant at *p* < 0.001. Question 1 (Q1) includes rankings one to six; Q2 includes rankings one to four; Q3, Q4, and Q5 includes rankings one to five. (*w*) denotes item worth in text.

**Table 6:** Results of rank analyses underscreened (N = 1570).

Item	Descriptive Statistics		Plackett–Luce Model Estimates	
	Item Ranked First (%)	Mean Rank	Item Worth Compared to the Average Worth (*w*) (95% CI)	Probability of Highest Rank (%)
(Q1) prefer to receive information about cervical cancer and screening from ^§^				
International public health agency	14.9	3.37	**1.12** (1.06; 1.18)	15.1
National public health agency	16.0	3.36	**1.11** (1.05; 1.18)	14.9
Provincial public health agency	25.9	2.39	**2.42** (2.29; 2.57)	32.6
Charities and non-profit organizations	2.7	4.33	**0.65** (0.61; 0.69)	8.7
Social media	3.1	4.90	**0.28** (0.26; 0.30)	3.7
A healthcare professional	37.5	2.66	**1.85** (1.75; 1.96)	24.9
(Q2) prefer to get the HPV test for my routine screening to prevent cervical cancer from ^§^				
Family physician	35.0	2.02	**1.73** (1.63; 1.83)	33.6
Gynaecologist	48.1	1.80	**2.26** (2.13; 2.40)	43.9
Nurse or nurse practitioner	15.5	2.62	**0.85** (0.81; 0.90)	16.6
Physician’s assistant	1.4	3.55	**0.30** (0.28; 0.32)	5.8
(Q3) prefer to receive the **invitation** to my routine screening to prevent cervical cancer by ^§^				
Postal mail	34.7	2.44	**1.53** (1.44; 1.62)	24.5
E-mail	39.6	1.92	**2.78** (2.62; 2.95)	44.7
Text message	7.4	3.29	**0.82** (0.77; 0.86)	13.1
Phone call	11.9	3.45	**0.66** (0.62; 0.69)	10.5
Online portal	6.4	3.90	**0.44** (0.41; 0.47)	7.1
(Q4) prefer to receive **reminders** about my routine screening to prevent cervical cancer by ^§^				
Postal mail	30.6	2.61	**1.32** (1.25; 1.39)	21.6
E-mail	39.8	1.94	**2.74** (2.58; 2.90)	44.8
Text message	12.9	3.08	**0.94** (0.89; 1.00) *	15.4
Phone call	10.6	3.44	**0.68** (0.65; 0.72)	11.2
Online portal	6.1	3.94	**0.43** (0.40; 0.46)	7.0
(Q5) prefer to receive the **results** of the HPV test by ^§^				
Postal mail	22.3	2.82	**1.15** (1.09; 1.22)	20.0
E-mail	34.6	2.05	**2.41** (2.28; 2.55)	41.8
Text message	5.8	3.49	**0.73** (0.69; 0.77)	12.6
Phone call	25.9	2.95	0.95 (0.90; 1.01)	16.5
Online portal	11.5	3.70	**0.52** (0.49; 0.55)	9.0

Note: ^§^ denotes significant Chi-square test (*p* < 0.001) showing non-random rankings. All estimates shown in bold are significant at *p* < 0.001, except for the estimate indicated with *, which is significant at *p* < 0.05. Question 1 (Q1) includes rankings one to six; Q2 includes rankings one to four; Q3, Q4, and Q5 includes rankings one to five; (*w*) denotes item worth in text.

## Data Availability

The data used in this study will not be published in a publicly available repository, in accordance with ethical requirements. The data will be available from the senior author (Z.R.) upon reasonable request, and upon agreement of confidentiality and data use policies provisioned by the primary institution.

## Abbreviations

The following abbreviations are used in this manuscript:

BC	British Columbia
BWS	Best–Worst Scaling
BWs	Best-Minus-Worst Score
CAD	Canadian Dollar
CI	Confidence Intervals
CPAC	Canadian Partnership Against Cancer
HCP	Healthcare Professional
HPV	Human Papillomavirus
PEI	Prince Edward Island
NWT	Northwest Territories
OR	Odds Ratios
stdBWs	Standardized Best-Minus-Worst Score
w	Worth

## Appendix A

STROBE Statement—checklist of items that should be included in reports of ***cross-sectional studies.***

	**Item No**	**Recommendation**	**Page** **No**
Title and abstract	1	(*a*) Indicate the study’s design with a commonly used term in the title or the abstract	1
		(*b*) Provide in the abstract an informative and balanced summary of what was done and what was found	1–2
**Introduction**			
Background/rationale	2	Explain the scientific background and rationale for the investigation being reported	2–4
Objectives	3	State specific objectives, including any prespecified hypotheses	4
**Methods**			
Study design	4	Present key elements of study design early in the paper	4–5
Setting	5	Describe the setting, locations, and relevant dates, including periods of recruitment, exposure, follow-up, and data collection	4–5
Participants	6	(*a*) Give the eligibility criteria, and the sources and methods of selection of participants	4–5
Variables	7	Clearly define all outcomes, exposures, predictors, potential confounders, and effect modifiers. Give diagnostic criteria, if applicable	5–6
Data sources/measurement	8 *	For each variable of interest, give sources of data and details of methods of assessment (measurement). Describe comparability of assessment methods if there is more than one group	5–6
Bias	9	Describe any efforts to address potential sources of bias	5
Study size	10	Explain how the study size was arrived at	5
Quantitative variables	11	Explain how quantitative variables were handled in the analyses. If applicable, describe which groupings were chosen and why	6
Statistical methods	12	(*a*) Describe all statistical methods, including those used to control for confounding	6–7
		(*b*) Describe any methods used to examine subgroups and interactions	6–7
		(*c*) Explain how missing data were addressed	5
		(*d*) If applicable, describe analytical methods taking account of sampling strategy	N/A
		(*e*) Describe any sensitivity analyses	N/A
**Results**			
Participants	13 *	(a) Report numbers of individuals at each stage of study—e.g., numbers potentially eligible, examined for eligibility, confirmed eligible, included in the study, completing follow-up, and analyzed	7
		(b) Give reasons for non-participation at each stage	7
		(c) Consider use of a flow diagram	7
Descriptive data	14 *	(a) Give characteristics of study participants (e.g., demographic, clinical, social) and information on exposures and potential confounders	7–8
		(b) Indicate number of participants with missing data for each variable of interest	7
Outcome data	15 *	Report numbers of outcome events or summary measures	8–12
Main results	16	(*a*) Give unadjusted estimates and, if applicable, confounder-adjusted estimates and their precision (e.g., 95% confidence interval). Make clear which confounders were adjusted for and why they were included	N/A
		(*b*) Report category boundaries when continuous variables were categorized	N/A
		(*c*) If relevant, consider translating estimates of relative risk into absolute risk for a meaningful time period	N/A
Other analyses	17	Report other analyses conducted—e.g., analyses of subgroups and interactions, and sensitivity analyses	12–13
Discussion			
Key results	18	Summarize key results with reference to study objectives	13
Limitations	19	Discuss limitations of the study, taking into account sources of potential bias or imprecision. Discuss both direction and magnitude of any potential bias	14–15
Interpretation	20	Give a cautious overall interpretation of results considering objectives, limitations, multiplicity of analyses, results from similar studies, and other relevant evidence	13–15
Generalizability	21	Discuss the generalizability (external validity) of the study results	14
Other information			
Funding	22	Give the source of funding and the role of the funders for the present study and, if applicable, for the original study on which the present article is based	15
* Give information separately for exposed and unexposed groups.			

## Appendix B

**Table A1:** Additional Best–Worst Scaling subgroup analyses for screening interval.

Item	Primary Language						Age			Ethnicity				
	EN (n = 2570)		FR (n = 631)		Other (n = 147)		≤30 (n = 408)	31–60 (n = 2161)	≥61 (n = 779)	North American Indigenous (n = 96)	North American Other (n = 1526)	European (n = 1029)	Asian (n = 446)	Other (n = 251)
**Attributes**														
Pap test	Ref													
HPV test	1.30		1.30		1.19 **		1.15	1.30	1.37	1.34	1.29	1.34	1.27	1.17 **
Pap and HPV test	2.04		1.68		1.80		1.68	2.06	1.85	2.28	1.96	2.04	1.86	1.68
Self-sampling	1.45		1.50		1.13 *		1.34	1.47	1.41	1.81	1.43	1.61	1.31	1.03 +
**Attribute levels for *Pap test***														
Every 3 years	1.85		1.93		2.24		1.61	1.90	2.00	1.64	1.96	1.77	1.68	2.55
Every 5 years	Ref													
Every 10 years	0.46		0.43		0.40		0.55	0.45	0.42	0.54	0.43	0.47	0.53	0.36
**Attribute levels for *HPV test***														
Every 3 years	1.79		1.81		1.88		1.43	1.84	1.89	1.78	1.81	1.78	1.66	2.10
Every 5 years	Ref													
Every 10 years	0.50		0.48		0.45		0.61	0.49	0.45	0.54	0.48	0.49	0.56	0.42
**Attribute levels for *Pap and HPV test (co-test)***														
Every 3 years	2.46		2.34		3.29		1.80	2.53	2.73	2.12	2.53	2.33	2.27	3.31
Every 5 years	Ref													
Every 10 years	0.32		0.34		0.23		0.40	0.32	0.28	0.38	0.30	0.32	0.37	0.28
**Attribute levels for *self-sampling***														
Every 3 years	2.00		2.00		2.38		1.77	2.01	2.16	1.71	2.08	1.97	1.83	2.29
Every 5 years	Ref													
Every 10 years	0.39		0.37		0.33		0.46	0.38	0.35	0.49	0.37	0.39	0.45	0.33
**Item**	**Living in Canada >10 years**						**Gender Identity**			**Income**				
	**Yes** **(n = 3135)**			**No** **(n = 213)**			**Woman** **(n = 3315)**		**Gender diverse** **(n = 33)**	**CAD ≤39,999** **(n = 836)**		**CAD 40,000–79,999** **(n = 1119)**	**CAD ≥80,000** **(n = 1393)**	
**Attributes**														
Pap test	Ref													
HPV test	1.30			1.24			1.29		1.32 *	1.28		1.23	1.36	
Pap and HPV test	1.96			1.90			1.95		2.23	1.80		1.90	2.11	
Self-sampling	1.46			1.15 **			1.44		1.55	1.34		1.44	1.51	
**Attribute levels for *Pap test***														
Every 3 years	1.86			2.16			1.89		1.50 **	1.68		1.87	2.04	
Every 5 years	Ref													
Every 10 years	0.46			0.40			0.45		0.54	0.51		0.46	0.42	
**Attribute levels for *HPV test***														
Every 3 years	1.78			2.11			1.80		1.48 **	1.65		1.77	1.91	
Every 5 years	Ref													
Every 10 years	0.50			0.44			0.49		0.61	0.54		0.50	0.46	
**Attribute levels for *Pap and HPV test (co-test)***														
Every 3 years	2.43			3.01			2.47		1.84	2.20		2.39	2.72	
Every 5 years	Ref													
Every 10 years	0.32			0.27			0.32		0.47	0.37		0.31	0.30	
**Attribute levels for *self-sampling***														
Every 3 years	1.98			2.46			2.02		1.58 **	1.87		1.96	2.15	
Every 5 years	Ref													
Every 10 years	0.39			0.34			0.38		0.56	0.43		0.39	0.35	

Note: + denotes not significant OR; * *p* < 0.05; ** *p* < 0.01; for all other OR, *p* < 0.001.

## Appendix C

**Table A2:** Additional Best–Worst Scaling subgroup analyses for age of screening initiation.

Item	Primary Language						Age			Ethnicity				
	EN (n = 2570)		FR (n = 631)		Other (n = 147)		≤30 (n = 408)	31–60 (n = 2161)	≥61 (n = 779)	North American Indigenous (n = 96)	North American-Other (n = 1526)	European (n = 1029)	Asian (n = 446)	Other (n = 251)
**Attributes**														
Pap test	Ref													
HPV test	1.33		1.34		1.16 **		1.12 **	1.34	1.39	1.17 *	1.35	1.40	1.22	1.09 +
Pap and HPV test	2.24		1.93		2.00		1.68	2.29	2.11	2.06	2.26	2.28	1.85	1.91
Self-sampling	1.41		1.49		0.99 +		1.19	1.45	1.40	1.60	1.44	1.59	1.18	0.94+
**Attribute levels for *Pap test***														
21 years	1.73		1.57		1.51		1.53	1.71	1.73	1.59	1.79	1.79	1.25	1.74
25 years	Ref													
30 years	0.52		0.59		0.58		0.58	0.53	0.52	0.58	0.51	0.50	0.69	0.54
**Attribute levels for *HPV test***														
21 years	1.73		1.61		1.47		1.44	1.71	1.81	1.49	1.80	1.78	1.26	1.77
25 years	1.09		1.07 *		1.06 +		1.14 **	1.08	1.06 +	1.07 +	1.07 **	1.10	1.13 **	1.02 +
30 years	Ref													
**Attribute levels for *Pap and HPV test (co-test)***														
21 years	2.12		1.86		2.15		1.74	2.07	2.25	1.75	2.24	2.15	1.44	2.33
25 years	1.16		1.11 **		1.21 **		1.28	1.14	1.14	1.13 +	1.11	1.21	1.19	1.11 *
30 years	Ref													
**Attribute levels for *self-sampling***														
21 years	1.65		1.56		1.33		1.42	1.63	1.70	1.38	1.73	1.70	1.18	1.74
25 years	1.24		1.19		1.39		1.29	1.22	1.26	1.11 +	1.22	1.26	1.25	1.27
30 years	Ref													
**Item**	**Living in Canada >10 Years**						**Gender Identity**			**Income**				
	**Yes** **(n = 3135)**			**No** **(n = 213)**			**Woman** **(n = 3315)**		**Gender diverse** **(n = 33)**	**CAD ≤39,999** **(n = 836)**		**CAD 40,000–79,999** **(n = 1119)**	**CAD ≥80,000** **(n = 1393)**	
**Attributes**														
Pap test	Ref													
HPV test	1.33			1.21			1.32		1.41 **	1.31		1.27	1.37	
Pap and HPV test	2.17			2.09			2.16		1.94	2.04		2.07	2.32	
Self-sampling	1.43			1.07 +			1.40		1.56	1.30		1.41	1.46	
**Attribute levels for *Pap test***														
21 years	1.70			1.55			1.69		1.24 +	1.52		1.66	1.84	
25 years	Ref													
30 years	0.53			0.55			0.53		0.60	0.61		0.55	0.48	
**Attribute levels for *HPV test***														
21 years	1.71			1.50			1.70		1.39 *	1.55		1.71	1.77	
25 years	1.09			1.06 +			1.08		1.10 +	1.07 *		1.08 **	1.10	
30 years	Ref													
**Attribute levels for *Pap and HPV test (co-test)***														
21 years	2.07			1.97			2.08		1.31 +	1.91		2.01	2.22	
25 years	1.16			1.12 *			1.15		1.23 +	1.10 **		1.18	1.17	
30 years	Ref													
**Attribute levels for *self-sampling***														
21 years	1.64			1.37			1.62		1.44 **	1.46		1.61	1.73	
25 years	1.23			1.26			1.23		1.18 +	1.17		1.23	1.28	
30 years	Ref													

**Note:** + Not significant OR; * *p* < 0.05; ** *p* < 0.01; for all other OR, *p* < 0.001.

## Appendix D

**Table A3:** Additional subgroup rank analyses using Placket–Luce models (item worth).

Item	Primary Language						Age					Ethnicity							
	EN		FR		Other		≤30		31–60		≥61	North American Indigenous	North American-Other	European		Asian		Other	
(Q1) prefer to receive information about cervical cancer and screening from																			
International public health agency	0.96 ^§^		1.99		1.18 ^§^		1.25		1.07 **		0.98 ^§^	0.95 ^§^	1.18	0.91 **		1.16 **		1.10 ^§^	
National public health agency	1.97		0.14		1.74		1.35		1.22		1.04 ^§^	1.43 **	0.80	1.75		2.09		1.23 **	
Provincial public health agency	2.60		5.29		2.45		2.13		2.51		3.02	2.01	2.66	2.80		2.38		2.47	
Charities and non-profit organizations	0.51		0.85		0.50		0.57		0.61		0.68	0.57	0.71	0.55		0.47		0.57	
Social media	0.13		1.31		0.20		0.27		0.24		0.22	0.26	0.35	0.14		0.17		0.26	
A healthcare professional	3.19		0.63		1.96		1.81		2.04		2.18	2.48	1.61	2.93		2.20		2.01	
(Q2) prefer to get the HPV test for my routine screening to prevent cervical cancer from																			
Family physician	2.15		2.30		1.82		1.67		2.18		2.43 ^§^	2.04	2.34	2.05		2.19		1.71	
Gynaecologist	2.13		2.12		3.85		3.06		2.30		1.63 ^§^	1.99	1.99	2.00		3.06		3.58	
Nurse or nurse practitioner	0.82		0.82		0.52		0.70		0.81		0.84	0.81 ^§^	0.85	0.87		0.60		0.63	
Physician’s assistant	0.27		0.25		0.28		0.28		0.25		0.30	0.30	0.25	0.28		0.25		0.26	
(Q3) prefer to receive the **invitation** to my routine screening to prevent cervical cancer by																			
Postal mail	1.49		1.43		1.03 ^§^		0.91 ^§^		1.36		2.55	1.18 ^§^	1.59	1.66		1.15 **		0.90 ^§^	
E-mail	2.79		2.42		3.29		3.12		2.73		2.61	2.18	2.51	2.88		3.34		3.01	
Text message	0.88		0.66		1.08 ^§^		1.21		0.90		0.55	0.89 ^§^	0.78	0.81		0.98 ^§^		1.07 ^§^	
Phone call	0.70		1.01		0.60		0.63		0.72		0.82	0.95 ^§^	0.82	0.66		0.62		0.82 **	
Online portal	0.39		0.44		0.45		0.46		0.41		0.33	0.46	0.40	0.39		0.43		0.42	
(Q4) prefer to receive **reminders** about my routine screening to prevent cervical cancer by																			
Postal mail	1.26		1.33		0.81 *		0.81		1.18		2.01	1.10 ^§^	1.38	1.35		1.01 ^§^		0.82 **	
E-mail	2.79		2.47		3.35		2.92		2.74		2.69	2.13	2.52	3.00		3.06		3.00	
Text message	1.04 ^§^		0.71		1.37		1.57		1.05 *		0.64	1.00 ^§^	0.90	0.95 ^§^		1.25		1.17 *	
Phone call	0.72		1.01 ^§^		0.64		0.64		0.75		0.87	0.95 ^§^	0.84	0.68		0.63		0.86 *	
Online portal	0.38		0.42		0.42		0.42		0.40		0.33	0.45	0.38	0.38		0.41		0.41	
(Q5) prefer to receive the **results** of the HPV test by																			
Postal mail	1.18		1.26		0.99 ^§^		0.85 **		1.11		1.85	1.09 ^§^	1.22	1.27		1.07 ^§^		0.92 ^§^	
E-mail	2.37		2.11		3.66		2.63		2.41		2.12	1.96	2.13	2.42		3.16		2.77	
Text message	0.76		0.58		0.92 ^§^		0.98 ^§^		0.76		0.56	0.83 ^§^	0.69	0.68		0.88 *		0.88 ^§^	
Phone call	0.95 *		1.39		0.61		0.90 ^§^		0.96 ^§^		1.13 **	1.24 ^§^	1.19	0.92 *		0.69		0.90 ^§^	
Online portal	0.49		0.46		0.49		0.50		0.51		0.40	0.45	0.47	0.52		0.49		0.49	
**Item**	**Living in Canada >10 Years**										**Gender Identity**		**Income**						
	**Yes**					**No**					**Woman**	**Gender diverse**	**CAD ≤39,999**	**CAD 40,000–79,999**			**CAD ≥80,000**		
(Q1) prefer to receive information about cervical cancer and screening from																			
International public health agency	1.05 *					1.45					1.07	1.33 ^§^	1.05 ^§^	1.08 *			1.08 *		
National public health agency	1.16					1.68					1.19	2.06	1.12 **	1.08 *			1.34		
Provincial public health agency	2.59					2.13					2.55	3.33	2.40	2.50			2.71		
Charities and non-profit organizations	0.63					0.49					0.62	0.51 **	0.64	0.68			0.57		
Social media	0.24					0.24					0.24	0.18	0.25	0.28			0.21		
A healthcare professional	2.07					1.68					2.05	1.19 ^§^	2.20	1.81			2.16		
(Q2) prefer to get the HPV test for my routine screening to prevent cervical cancer from																			
Family physician	2.22					1.48					2.18	0.99 ^§^	2.13	2.06			2.26		
Gynaecologist	2.09					5.01					2.16	4.06	2.31	2.14			2.12		
Nurse or nurse practitioner	0.82					0.52					0.80	1.18 ^§^	0.74	0.84			0.81		
Physician’s assistant	0.26					0.26					0.27	0.21	0.27	0.27			0.26		
(Q3) prefer to receive the **invitation** to my routine screening to prevent cervical cancer by																			
Postal mail	1.53					0.77					1.45	1.30 ^§^	1.56	1.43			1.40		
E-mail	2.68					3.59					2.73	2.90	2.50	2.76			2.86		
Text message	0.81					1.31					0.84	0.90 ^§^	0.73	0.83			0.92 **		
Phone call	0.74					0.68					0.74	0.63 *	0.87	0.74			0.68		
Online portal	0.40					0.41					0.41	0.47	0.41	0.41			0.40		
(Q4) prefer to receive **reminders** about my routine screening to prevent cervical cancer by																			
Postal mail	1.31					0.69					1.25	0.81 ^§^	1.37	1.28			1.16		
E-mail	2.70					3.38					2.74	2.56	2.39	2.80			2.94		
Text message	0.95 **					1.65					0.98 ^§^	1.22 ^§^	0.82	0.97 ^§^			1.10 **		
Phone call	0.76					0.71					0.76	0.66 ^§^	0.93^§^	0.74			0.70		
Online portal	0.39					0.37					0.39	0.60 *	0.40	0.39			0.38		
(Q5) prefer to receive the **results** of the HPV test by																			
Postal mail	1.22					0.77					1.19	0.98 ^§^	1.31	1.17			1.14		
E-mail	2.30					3.64					2.35	2.54	2.10	2.41			2.48		
Text message	0.71					1.12 ^§^					0.73	0.66 *	0.69	0.74			0.75		
Phone call	1.02 ^§^					0.73					0.99 ^§^	0.93 ^§^	1.21	0.98 ^§^			0.90		
Online portal	0.49					0.44					0.49	0.65 ^§^	0.43	0.49			0.53		

**Note:** ^§^ Non-significant *p*-value; * *p* < 0.05; ** *p* < 0.01; all other *p*-values < 0.001.
